# Evaluation of the antibacterial activity of a new ozonized olive oil against oral and periodontal pathogens

**DOI:** 10.4317/jced.54929

**Published:** 2018-11-01

**Authors:** Giampiero Pietrocola, Matteo Ceci, Francesco Preda, Claudio Poggio, Marco Colombo

**Affiliations:** 1 Department of Molecular Medicine, Unit of Biochemistry, University of Pavia, Pavia, Italy; 2 Department of Clinical-Surgical, Diagnostic and Pediatric Sciences – Section of Dentistry, University of Pavia, Pavia, Italy

## Abstract

**Background:**

In the present study, the antimicrobial properties of a new ozonized olive oil (O-zone gel) against oral and periodontal pathogens will be evaluated and compared with that of common CHX-based agents.

**Material and Methods:**

O-zone gel was compared with two agents based on chlorhexidine digluconate (CHX): Corsodyl Dental Gel and Plak Gel. A. actinomycetemcomitans, P. intermedia and S. mutans, were selected and the antibacterial capability of the compounds was tested by using direct contact agar diffusion test (DCT) and minimum inhibitory concentration (MIC) and minimum bactericidal concentration (MBC) evaluations. Differences between specific means were analyzed by a one-way analysis of variance (ANOVA). Group means were compared using a one-way ANOVA and Tukey’s test (*P*<0.05).

**Results:**

O-zone gel reported inhibition zones which correspond to 33% and 43% of that achieved by the CHX agents. No inhibition of bacterial growth (MIC) on the Gram-positive strain by using O-zone gel was found and no antimicrobial effect (MBC) was observed by using O-zone gel on both Gram-negative and -positive strains.

**Conclusions:**

The new ozonated oil was a relatively moderate antiseptic. Gram-negative bacteria proved to be more sensitive to ozonized olive oil than Gram-positive ones. The ozonized olive oil demonstrated a lower antibacterial activity if compared to the CHX-based agents tested.

** Key words:**Agar diffusion test, antibacterial activity, direct contact test, ozone, ozonized olive oil.

## Introduction

In the last few decades, the usage of ozone in dentistry has been proposed because of its antimicrobial, biocompatible and healing properties. In the last years a number of therapeutic protocols with ozone have been developed to address dental infections associated with periodontal disease and caries (1,2).

Various studies have evaluated the use of ozone for many dental therapies such as: bone regeneration (3), remineralization of white spot lesions (4,5), endodontic treatments (6), periodontal pocket disinfection (7), tooth bleaching and management of tooth sensitivity (8,9), TMJ pain control (10).

Three different forms of ozone are basically used: ozone gas, ozonated water and ozonated oil. Ozonated oils like ozonated sunflower oil, olive oil and groundnut oil are capable of inducing the reduction of many oral microorganisms (11).

Plaque biofilm is the main cause of both caries and periodontal disease. Ozone has been demonstrated to be useful to control oral infectious microorganisms in dental plaque (12). Ozonated oil has been recently used as an alternative in patients with periodontal disease by using it as a sub-gingival irrigant. The antimicrobial property of Ozone demonstrated to be effective in reducing the number of various periodontal bacteria; however it was not successful in completely eliminating these bacteria embedded in the plaque biofilm (13).

Ozone therapy has been also proposed as a new method for treating caries. It has been suggested that the application of ozone to carious lesions will arrest or reverse these lesions and that the use of ozone can kill bacteria present within carious lesion, painlessly and even without anesthetic (14). Ozone seems to exert its antimicrobial action through the synergistic action of damaging the cytoplasmic membrane of cells and of inducing the modification of intracellular contents because of secondary oxidants effects (15). This action is non-specific and selective to microbial cells (16).

O-zone gel (Alnitec, Cremosano, Cr, Italy) is a new ozonized olive oil with bactericidal and fungicidal properties whose use is suggested for periodontal treatments and for early caries therapy.

In the present study, the antimicrobial properties of this new ozonized olive oil against oral and periodontal pathogens will be evaluated and compared with that of common antimicrobial gel-formulated agents (Corsodyl Dental Gel and Plak Gel) by using the direct contact agar diffusion test (DCT) and minimum inhibitory concentration (MIC) and minimum bactericidal concentration (MBC) evaluation. The null hypothesis of the study was that the ozonized olive oil did not de¬monstrate antibacterial effects; therefore, that there was no difference between the antibacterial capabilities of the three different antimicrobial agents tested.

## Material and Methods

A new ozonized olive oil was selected for this study: O-zone gel (Alnitec, Cremosano, Cr, Italy). Its antibacterial activity was compared with two common antimicrobial agents based on chlorhexidine digluconate (CHX): Corsodyl Dental Gel (GSK, Brentford, Middlesex, UK) and Plak Gel (Polifarma, Roma, Italy).

Microorganisms and media. *Aggregatibacter actinomycetemcomitans* (ATCC number: 33384), *Prevotella intermedia* (ATCC number: 25611) and *Streptococcus mutans* (ATCC number: 25175), were used in the study. *A. actinomycetemcomitans* was cultured in brain heart infusion (BHI) broth, *P. intermedia* was cultured in tryptic soy broth (TSB), containing 5% sheep blood, 0.5% vitamin K and *S. mutans* was cultured in TSB. The bacteria were inoculated by loop transfer from frozen tubes into 3 mL slant nutrient broth and grown at 37°C for 24 h under appropriate (aerobic/anaerobic) conditions. Bacteria from these cultures were transferred onto an appropriate solid medium and incubated overnight. Selected colonies were transferred to the appropriate liquid medium and were incubated for 4–6 h to achieve log phase growth. The bacterial suspension was centrifuged, rinsed twice using PBS and suspended to the appropriate bacterial density by comparing the OD600 of the sample with a standard curve relating OD600 to cell number.

Direct contact agar diffusion tests. For direct contact agar diffusion tests, 5 mL of fresh broth agar were prepared in 6-cm Petri dishes, and bacteria were spread at 5×105 cfu on the broth agar surface. Thereafter, four wells (for each material to be tested) of 3 mm in diameter and 2 mm in depth were made with a punch by removing the agar at equidistant points and then filled immediately with the materials to be evaluated. All plates were maintained at room temperature for 2 h for pre-diffusion of the materials and then incubated for 24–96 h at 37°C. The inhibition zone around each well was measured in two perpendicular locations with a millimeter ruler (sliding calipers) with accuracy of 0.5 mm. The size of the inhibition zone was calculated as follows.

Size of inhibition zone = (diameter of halo – diameter of specimen) x ½

Antibacterial efficiency, minimum inhibitory concentration (MIC) and minimum bactericidal concentration (MBC). To assess the susceptibility of oral pathogenic bacteria to tested compounds, Minimum Inhibitory Concentrations (MIC) and Minimal Bactericidal Concentrations (MBC) were determined. For this purpose, 2 μl of 106 cfu/mL were added to 2 mL of fresh broth containing serial dilution (10 - 0.01%) of the tested compounds. The cultures were incubated at 37°C at 200 rpm for 24 h. Tubes showing no visible turbidity were considered to represent the MIC and were subsequently inoculated onto sterile 6 cm nutrient agar plates and incubated for 24 h. The lowest concentration at which no growth was observed was considered to be the MBC (Shih YH, Chang KW, Hsia SM, Yu CC, Fuh LJ, Chi TY, *et al.* *In vitro* antimicrobial and anticancer potential of hinokitiol against oral pathogens and oral cancer cell lines. Microbiol Res. 2013; 168: 254±262. doi: 10.1016/j.micres.2012.12.007 PMID: 23312825).

Statistical methods. All of the assays were performed in triplicate. Differences between specific means were analyzed by a one-way analysis of variance (ANOVA). Group means were compared using a one-way ANOVA and Tukey’s test. The data are shown as the means ± standard deviation (SD). Differences between the variants were considered significant when *P* < 0.05.

## Results

**Figure 1:**
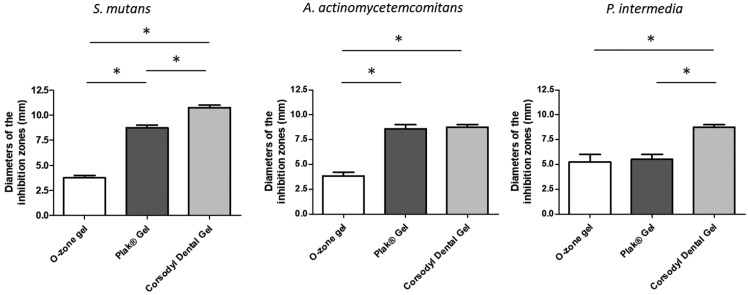
Antibacterial activities of a new ozonized olive oil comparing to two common CHX-based agents. The antibacterial effect was assesed by direct contact agar diffusion test against three oral pathogenic bacterial strains. Means and SD of results of two independent experiments, each performed in triplicate, are presented. Statistically significant difference is indicated (*P< 0.05).

Agar diffusion assay. The antibacterial effect of O-zone gel, Corsodyl Dental Gel® and Plak Gel® for the selected oral pathogenic bacterial strains was determined by measuring the radius of the inhibition zone. This test showed that all compounds were able to inhibit the growth of both Gram-negative and -positive bacterial strains used. Diameters of the inhibition zones and standard deviations are shown in  Table 1 and Figure 1. Treatment with O-zone gel led to inhibition zone of 3.5 mm on both *S. mutans* and *A. actinomycetencomitans*, whereas the inhibition zone achieved by the CHX agents was significantly higher (*P*< 0.05). The inhibition zone caused by O-zone gel on *P. intermedia* was of 4.5 mm (~1.3 times more than that observed on *S. mutans* and *A. actinomycetencomitans*), whereas the inhibition zone achieved just by Corsodyl Dental Gel was significantly higher (*P*< 0.05). No statistical significant differences between O-zone gel and Plak Gel on *P. intermedia* growth was observed. Corsodyl Dental Gel showed significantly higher (*P*< 0.05) inhibition zone than Plak Gel on both *S. mutant* and *P. intermedia*.

**Table 1:**
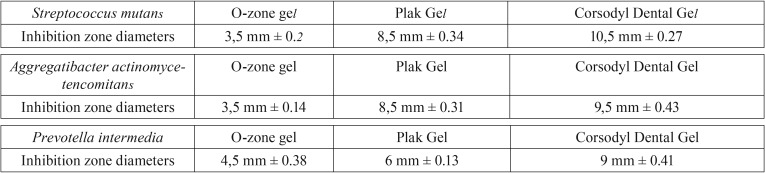
Mean diameter ± standard deviation (mm) of the bacterial inhibition zone by common antimicrobial gel-formulated agents evaluated by Direct contact agar diffusion test. All the assays were conducted in triplicate and the results were recorded in terms of the average diameter of inhibition zone (mm).

MIC and MBC of the tested compounds. The obtained MIC/MBC values of O-zone gel, Corsodyl Dental Gel and Plak Gel for the selected bacterial strains are shown in  Table 2. All the tested compounds showed anti-bacterial activity on Gram-negative bacterial strains (*A. actinomycetemcomitans* and *P. intermedia*) although with different concentrations. Both strains had similar MIC (10%) by using O-zone gel, otherwise the MIC values found by using Corsodyl Dental Gel or Plak Gel (≤ 0.01 – 1% respectively) were significant lower (*P* < 0.05). No inhibition of bacterial growth on the Gram-positive strain used (*S. mutans*) by using O-zone gel was found. Conversely, both Corsodyl Dental Gel and Plak Gel were able to inhibit the *S. mutans* cells growth with a MIC value of ≤ 0.01 and 1% respectively. No antimicrobial effect (MBC) was observed by using O-zone gel on both Gram-negative and -positive strains. Conversely, both Corsodyl Dental Gel and Plak Gel were able to cause the complete bacterial suppression of all strains used (MBC values ranging from≤ 0.01 to 10%). The results of the inhibition zone and MIC/ MBC experiments were consistent. Corsodyl Dental Gel and Plak Gel were strong antiseptics; O-zone Gel was relatively moderate antiseptic.

**Table 2:**
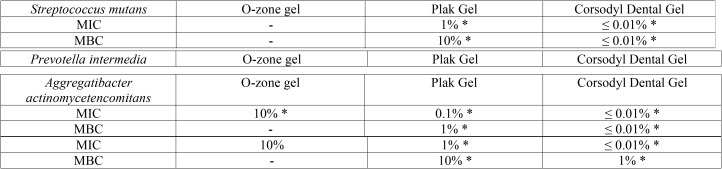
Minimal Inhibitory Concentration (MIC) and Minimal Bactericidal Concentration (MBC) of the tested compounds on oral pathogenic bacterial strains. * Indicate statistical significant differences between values in the same row (*p*< 0.05).

## Discussion

Ozone seems to strongly inhibit the formation of dental plaque and reduce the number of pathogens both Gram-positive and Gram-negative organism (17). The oxidative power of ozone is 1.5 times greater than that of chloride when used as an antimicrobial agent (1). This effect of oxidation gives to ozone its bactericidal, virucidal, and fungicidal activity (12). According to microbiological studies, ozone is capable of killing Gram-positive and Gram-negative bacteria, including Pseudomonas aeruginosa and Escherichia coli (1). This antimicrobial capacity is the result of ozone effects on cells such as damaging the cytoplasmic membrane due to ozonolysis of dual bonds and inducing changes of cytoplasmic contents. This action seems not to damage human body cells; the reason attributed to this is the antioxidant ability of mammalian cells (18).

In our study the antibacterial properties of ozonized olive oil were evaluated with direct contact agar diffusion test (DCT). The agar diffusion test (ADT) has been widely used to investigate the antimicrobial activity of dental materials and it is one of the most common and simplex methods. However, it has some limitations such as lack of standardization of inoculum density, adequate culture medium, agar viscosity, plates storage condition and dependency on the solubility and diffusion characteristic of both the test material and media (19). On the contrary, the DCT mimics the direct contact between microorganisms and the antimicrobial gel and it has several advantages such as reproducibility and quantitative assay (20).

MICs and MBCs of the antimicrobial agents investigated in the present study were determined using the micro dilution test method. MICs are defined as the lowest concentration of an antimicrobial that will inhibit the visible growth of a microorganism after an overnight incubation and are considered the gold standard for determining the susceptibility of organisms to antimicrobials. On the other hand, MBCs are the lowest concentrations of an antimicrobial that will prevent the growth of an organism following a subculture on antimicrobial-free media (21). MIC/MBCs are used for determining the potential resistance of an antimicrobial and making rational decisions in determining how successful an antimicrobial treatment is likely to be (21).

The bacterial strains selected for this study were *Aggregatibacter actinomycetemcomitans, Prevotella intermedia and Streptococcus mutans*. *A. actinomycetemcomitans* and *P. intermedia* are acknowledged as the main etiologic agents in the appearance of periodontal diseases (22). *S. mutans* is otherwise the predominant species isolated from human saliva and dental plaque and it has been widely recognized as the major etiologic agent for dental caries (23).

The null hypothesis of this study was rejected. In fact, the new ozonized olive oil demonstrated antibacterial effects and differences between the antibacterial capabilities of the three different antimicrobial agents tested were demonstrated.

In DCT the new ozonized olive oil showed antibacterial efficacy against all the bacterial species tested. However, except for *P. intermedia*, for which the inhibition zones led by O-zone gel and the CHX agents were similar, the antibacterial capability of the O-zone gel proved to be lower than that observed by Corsodyl Dental Gel and Plak Gel.

As regards the MIC/MBC values, all the tested compounds showed inhibition of bacterial growth (MIC) on Gram-negative bacterial strains (*A. actinomycetemcomitans* and *P. intermedia*) although with different concentrations, while no inhibition of bacterial growth on the Gram-positive strain (*S. mutans*) by using O-zone gel was found. These findings are in accordance with some studies that showed that Gram-negative bacteria, such as *P. intermedia* and *P. gingivalis* were more sensitive to ozone than Gram-positive oral streptococci (24).

Anyway, the DCT and MIC/MBC results of our study demonstrated a lower antibacterial activity of the ozonized olive oil if compared to the CHX compounds. In addition to this, no antimicrobial effect (MBC) was observed by using the O-zone gel on both Gram-negative and -positive strains. Conversely, both CHX agents were capable to obtain the suppression of all the strains tested.

Our results are not in accordance with some Authors that compared the effectiveness of ozone gel with that of CHX, against periodontal microorganisms, showing no significant differences in the antibacterial capability of ozone compared with that of 2% CHX (25). Another study compared the effect of periodontal pockets irrigation with ozonated gel or 0.2% CHX in patients with chronic periodontis, concluding that ozone application represents a stronger alternative to CHX and it may serve as good tool during supportive periodontal therapy (26). Furthermore, in discordance with our findings, Baysan proved that the number of Gram-positive bacteria in carious root lesions is considerably reduced by ozone therapy, and that the lesions clinically change, arresting its progression (27). Similarly, two other studies demonstrate a strong antimicrobial activity of ozone against *S. mutans* infections *in vitro* in bovine dentine as well as in ex vivo conditions (28,29)

However our results are similar to that reported in a recent Cochrane Review which identified 3 randomized controlled trials (RCTs): two of the three RCTs investigated the effect of ozone therapy on crown lesions, while the third investigated the effect on root lesions; providing no evidence that the application of ozone arrests the decay process and that ozone had a minimal effect on the viability of microorganisms organized in a cariogenic biofilm (30,31).

In conclusion, our study demonstrated that Corsodyl Dental Gel and Plak Gel were strong antiseptics, while the new ozonated oil (O-zone gel) was a relatively moderate antiseptic. Anyway, further studies represent a fundamental need for more evidence of appropriate rigor and quality before the use of ozone can be accepted as a viable antimicrobial agent into routinely dental therapies.

## Conclusions

Within the limitations of this *in vitro* study, the new ozonated oil was a relatively moderate antiseptic. Furthermore, the new ozonized olive oil (O-zone gel) demonstrated a lower antibacterial activity if compared to the CHX-based agents tested.
